# Wound Infiltration and Instillation Technique for Postoperative Analgesia Using Bupivacaine in Patients Undergoing Lumbar Spine Surgeries

**DOI:** 10.7759/cureus.23592

**Published:** 2022-03-28

**Authors:** Manoj Kumar, Saumya Srivastava, Dheer Singh, Jay Brijesh Singh Yadav, Vimal Kumar

**Affiliations:** 1 Anaesthesiology, Uttar Pradesh University of Medical Sciences, Saifai, IND; 2 Surgery, North Delhi Municipal Corporation (NDMC) Medical College and Hindu Rao Hospital, New Delhi, IND

**Keywords:** postoperative pain, infiltration, instillation, laminectomy, bupivacaine

## Abstract

Background: Pain relief after surgery continues to be a major medical challenge in clinical practice. Lumbar spine surgery is associated with significant postoperative pain. Providing optimal analgesia locally in the area of surgical wound, with little systemic side-effects, is a favourable option and has become an intrinsic part of multimodal analgesia. We aimed to assess and compare the effectiveness of local infiltration and instillation of bupivacaine for postoperative analgesia in patients undergoing lumbar spine surgery.

Materials and methods: Forty-four adult patients of the American Society of Anesthesiologists (ASA) class I and II were randomly assigned into two groups, incorporating 22 patients per group. After the completion of lumbar spine surgery and after hemostasis was achieved, patients in group A received instillation of 20 ml of 0.25% bupivacaine at the surgical wound site and patients in group B received 20 ml of 0.25% bupivacaine infiltration into the paravertebral muscles on either side. Postoperative numerical rating scale (NRS) pain scores at 1, 2, 3, 4, 5, 6, 7, 8, 14, 20, and 24 hours; the time to first analgesic required, total rescue analgesic consumption, and adverse effects were recorded. Statistical analysis was done using IBM SPSS Statistics for Windows, Version 20.0 (Released 2011; IBM Corp, Armonk, New York, United States).

Results: Time to the first analgesic requirement was significantly longer in group A (12.39±1.56 hours) compared to the B group (2.48±0.58 hours) (P < 0.001). The amount of rescue analgesia (diclofenac sodium) required was significantly higher in group B (135.00±46.17 milligrams) compared to A (93.75±33.32 milligrams) (P = 0.001). The number of analgesic demands was higher in the infiltration group compared to the instillation group and was observed to be statistically significant. Hemodynamic parameters remained comparable between the groups.

Conclusion: Local instillation of surgical wound site provided better pain control than infiltration technique and is effective and safe postoperative analgesia in patients undergoing laminectomy surgeries.

## Introduction

Lumbar spine surgery is a procedure performed in neurosurgical and orthopaedic practice. Patients usually experience severe postoperative pain after surgery [1]. Commonly performed spinal surgeries include laminectomies, discectomies, spinal fusions, instrumentations, scoliosis corrections, and spinal tumour excision. Optimal postoperative pain relief helps in early ambulation and induction of physiotherapy, gives satisfaction to the patients, prevents the development of chronic pain, and plays the principal role in decreasing morbidity and mortality [2,3]. Nowadays various postoperative analgesic options are available. Intravenous opioids, non-steroidal anti-inflammatory drugs (NSAIDs), intrathecal use of opioids, and local anaesthetics have been studied. Many of these techniques are restricted by high failure rates, high prices, challenges due to technical reasons, labour-intensive processes, toxic effects, and procedure-related complications [4 ].

Wound infiltration or instillation with local anaesthetics is a simple concept for providing effective postoperative analgesia for a variety of surgical procedures without any major side effects [5]. The wound infiltration technique acts by blocking the transmission of pain from nociceptive afferents directly from the wound surface and also decreases the local inflammatory response to injury. The infiltration of wounds with local anaesthetic drugs has become an appealing method in postoperative analgesia due to its safety, simplicity, and low cost [6,7]. It has been confirmed in several reports that infiltration of the wound with ropivacaine can significantly curtail postoperative pain, reduce supplemental analgesic requirement, as well as decrease the hospital stay following some surgeries like joint replacement, abdominal surgeries, and caesarean sections [8,9].

Local anaesthetic drug instillation into the wound dispenses postoperative analgesia in various surgical procedures like herniorrhaphy and laparoscopic cholecystectomy [10]. The simple technique of wound instillation with bupivacaine or ropivacaine and allowing a contact time of 60 seconds may decrease postoperative pain lumbar laminectomy. In wound instillation technique, the mechanism of pain relief could be due to the effect of local anaesthetic drug bupivacaine, which acts on the pain receptors distributed in the soft tissues and the nerve endings exposed in the wound area from the skin to the dura meninges (skin, paraspinal muscles, posterior longitudinal ligament, dorsal annulus, facet joint capsule, nerve root that was compressed and the spinal meninges, the dura, supplied by recurrent nerve of Von Luschka) [4].

There are very few recent studies regarding the use of infiltration with local anaesthetics for the relief of postoperative pain after lumbar spine surgical procedures [11,12,13]. The objective of this study was to assess and compare the effectiveness of local infiltration and instillation of bupivacaine for postoperative analgesia in patients undergoing lumbar laminectomies.

## Materials and methods

After obtaining approval of the Institutional Ethical Committee, Uttar Pradesh University of Medical Sciences, Saifai, Uttar Pradesh, India (678/UPUMS/Dean/2019-20/E.C./2019-18) and informed consent of the patients, this study was conducted in the Department of Anesthesia, Uttar Pradesh University of Medical Sciences in Saifai, Uttar Pradesh, India. The study was conducted in prospective randomized double-blind manner.

Inclusion and exclusion criteria

Patients in the American Society of Anesthesiologists (ASA) physical status classification I and II, aged between 30 and 60 years, having body mass index (BMI) of 18-29kg/m^2^, and scheduled to undergo single-level lumbar laminectomy under general anaesthesia were included in the study. Patients who did not give consent, were in ASA physical status classification III and IV, were scheduled for multiple distance or double-sided laminectomy, and had preceding lumbar disc surgery, neurological deficits, history of substance abuse, local anaesthesia allergy, bleeding, or cerebrospinal fluid leak, were excluded from the study. Considering alpha error of 0.05 and power of study as 95%, the estimated sample size comes out to be 22 patients per group. Patients were randomly assigned in two groups of 22 each, employing a computer-generated random number table. Group A (n=22) had 20 ml Bupivacaine (0.25%) diluted in normal saline instillation done at the surgical site and Group B (n=22) had 20 ml Bupivacaine 0.25% diluted in normal saline infiltration at the surgical wound site.

Pre-anaesthetic check-up was done for all the patients, details related to clinical history and general physical examination were noted, and all necessary investigations were carried out. Patients included in the study were assessed the day before surgery and were instructed how to judge the intensity of pain using a numerical rating scale (NRS), a scale of 0-10 (0 = no pain and 10 = worst pain). The study was a double-blind study, and the patients and the observer physician were blinded to the technique used. Upon arrival in the operating room, peripheral intravenous access was obtained with an 18 G cannula and lactated ringer`s solution was started at 6 ml/kg. All the standard ASA monitors were applied and non-invasive blood pressure (NIBP), heart rate (HR), electrocardiogram, peripheral oxygen saturation (SpO2) was recorded and carried out throughout the perioperative period. All patients were premedicated with injection glycopyrrolate (0.2mg), injection fentanyl (2mcg/kg), injection midazolam (0.25mg/kg) intravenously. After three minutes of preoxygenation, anaesthesia was induced with injection propofol 2mg/kg IV; injection succinylcholine 1.5mg/kg IV to facilitate endotracheal intubation. Anaesthesia was maintained using 67% nitrous oxide (N20) in 33% 02 and halothane 0.5% using controlled ventilation. Neuromuscular blockade was attained using vecuronium 0.08-0.12 mg/kg intravenously. Paracetamol 1 gram i.v. for intraoperative analgesia was used in both the groups

At the end of the surgical procedure and when hemostasis was achieved, Group A patients received instillation of 20 ml bupivacaine 0.25% into the surgical wound and Group B patients received 20 ml bupivacaine 0.25% infiltration into the paravertebral muscles on each side by the surgeon. The drug solution was allowed to remain in the wound for a contact time of 60 seconds. Thereafter, the wound was closed in layers and no mopping or suctioning was done. Tracheal extubation was done with reversal of the neuromuscular blockade done with injection neostigmine 0.05mg./kg and glycopyrrolate 0.01mg/kg body weight. When the patients were completely awake, an assessment for pain was done and recorded. Patients who remained sedated after one hour were not included in the study.

Postoperative pain was evaluated by an independent observer who was blinded to the study using a numeric rating scale, first at zero hours, i.e., immediately after extubation, and then at every one hour for the first eight hours and then at every six hours till 24 hours. The total duration of analgesia was considered from the time the study drug was instilled or infiltrated to the time the patient required rescue analgesia for the first time. When pain score reached ≥ 4 points on the numeric rating scale, injection diclofenac sodium 75 mg deep intramuscular was administered as rescue analgesia with lockout period of eight hours and 225 mg as maximum dose in 24 hours. The total amount of analgesic demand in 24 hours and hemodynamic parameters like HR, mean arterial pressure (MAP), and respiratory rate (RR) were recorded after the block and patients were monitored up to 24 hours after the block.

Statistical analysis

Quantitative variables were expressed as Mean±SD and compared between groups using an unpaired t-test. Qualitative variables were expressed in terms of frequencies and compared between groups using the Chi-square test/Fisher's exact test. A p-value <0.05 was considered statistically significant. The data was stored in a Microsoft Excel spreadsheet (Microsoft Corporation, Redmond, Washington, United States) and statistical analysis was performed using IBM SPSS Statistics for Windows, Version 20.0 (Released 2011; IBM Corp, Armonk, New York, United States).

## Results

**Figure 1:**
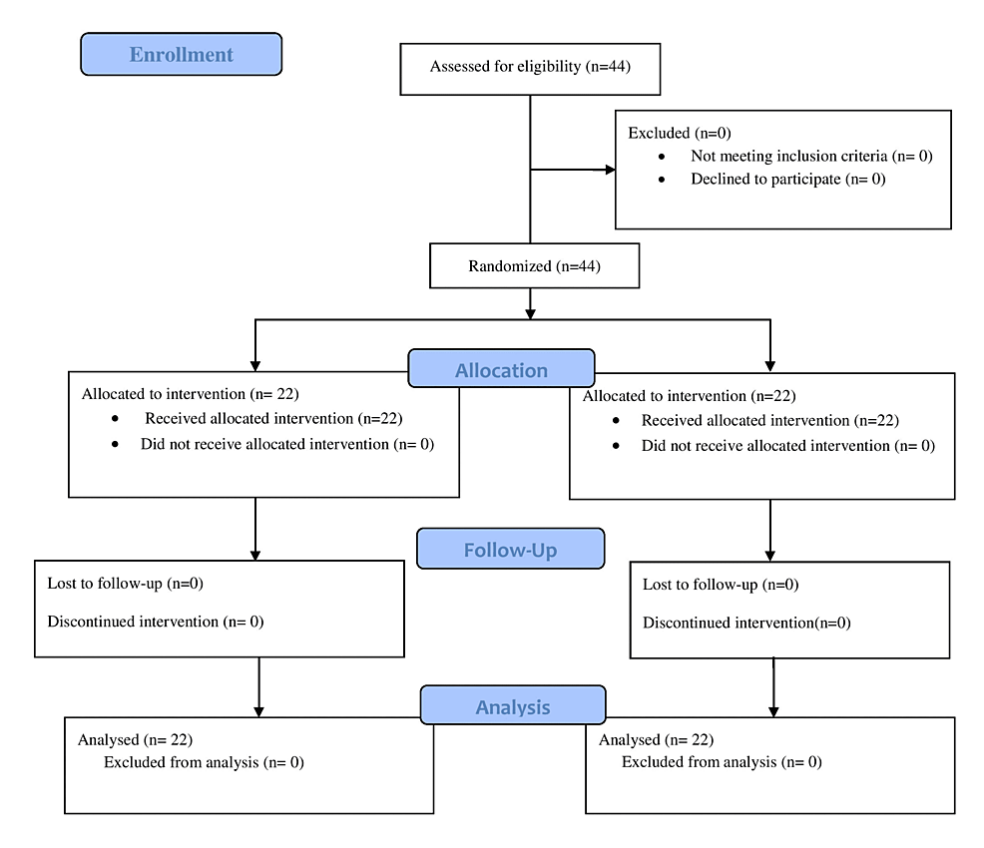
CONSORT flow diagram CONSORT: Consolidated Standards of Reporting Trials

In this clinical trial, 44 patients undergoing single-level lumbar laminectomy were enrolled for the study. In group A, 22 patients received wound instillation with bupivacaine and another 22 patients received wound infiltration with bupivacaine in group B. No patients were excluded from our study (Figure 1). 

The demographic characteristics (age, weight, height, BMI, gender, and ASA physical status classification) were comparable between the groups (p>0.05) (Table 1). The mean duration of analgesia was higher in group A (12.39±1.56 hours) compared to group B (2.48±0.58 hours). The mean values were statistically significant between the groups (P<0.001) (Table 1). The amount of analgesia required in group A and group B was 93.75±33.32 and 135.00±46.17mg respectively. The mean values were observed to be higher in group B compared to group A and were statistically significant among the groups (P<0.05) (Table 1).

**Table 1:** Distribution of demographic data among the studied groups ASA: American Society of Anesthesiologists

Characteristics	Group A (n=22) Mean±SD	Group B (n=22) Mean±SD	*P value
Age (years)	40.45±10.07	37.80±10.75	0.23
Weight (kg)	66.40±8.13	64.70±7.03	0.242
Height (cm)	168.65±9.11	170.65±7.05	0.221
BMI (kg/m^2)^	23.31±1.75	22.27±2.65	0.076
Sex (Male)	13	12	0.372
Sex (Female)	7	8	
ASA Grade I	16	13	0.144
ASA Grade II	4	7	
Duration of surgery (minutes)	147.70±17.21	150.25±16.02	0.35
Duration of analgesia (hours)	12.39±1.56	2.48±0.58	<0.001
Total amount of analgesic required (milligrams)	93.75±33.32	135.00±46.17	0.001
Number of analgesic demands			
1	15 (75%)	6 (30%)	0.002
2	5 (25%)	12 (60%)	0.013
3	0	2 (10%)	0.073

The demands of rescue analgesia in group A were one time in 15 (75%) patients and two times in five (25 %) patients. In group B, it was one time in six (30%) patients and two times in 12 (60%) patients (Table 1). The number of demands were higher in the infiltration group compared to the instillation group and was observed to be statistically significant in both groups.

The changes in the NRS score at 1, 2, 4, 5, 6, 7, 8, 14, 20 and 24 hours after completion of surgery are given in Table 2. The mean NRS values were comparable between the groups at all time periods except at three hours and eight hours where a statistically higher NRS score was observed in group B (p=0.001). The mean NRS values were higher in group B compared to group A, except after 20 hours where it was higher in group A but not statistically significant (p>0.05).

**Table 2:** Comparison of Numerical Rating Scale (NRS) among the studied groups

Numerical Rating Scale (NRS)	Group A (n=22) Mean±SD	Group B (n=22) Mean±SD	*P value (Unpaired t-test)
Baseline (0 minute)	0.00±0	0.00±0	-
1 hour	0.55±0.51	0.60±0.50	0.378
2 hours	0.65±0.49	1.00±0.86	0.061
3 hours	0.85±0.37	3.90±1.02	0.001
4 hours	1.0±0.00	1.30±1.13	0.121
5 hours	1.20±0.41	1.20±1.14	0.50
6 hours	1.30±0.47	1.80±1.61	0.095
7 hours	1.80±0.41	1.80±1.70	0.50
8 hours	2.05±0.39	2.75±1.45	0.022
14 hours	3.75±0.85	1.70±1.03	0.001
20 hours	1.85±1.18	1.80±0.52	0.432
24 hours	2.00±1.26	1.70±0.92	0.197

**Figure 2:**
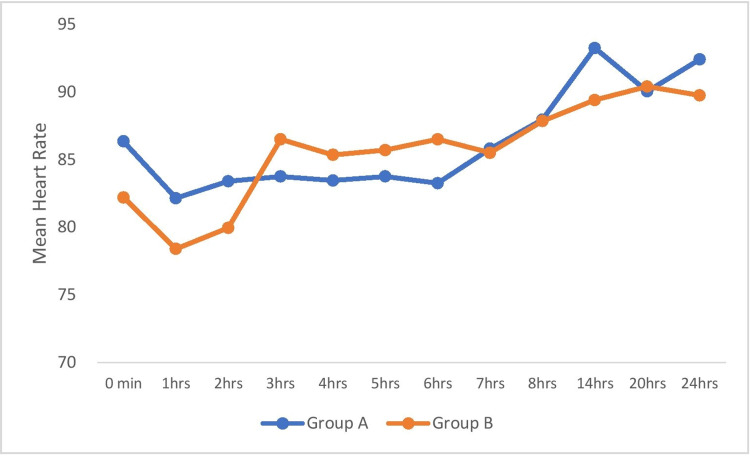
Comparison of mean heart rate (beats per minute) among the groups across the time periods

Figure 2 shows the comparison of HR among the groups across the time periods. There was no statistically significant difference in HR among the groups at all the time periods (p=0.52).

**Figure 3:**
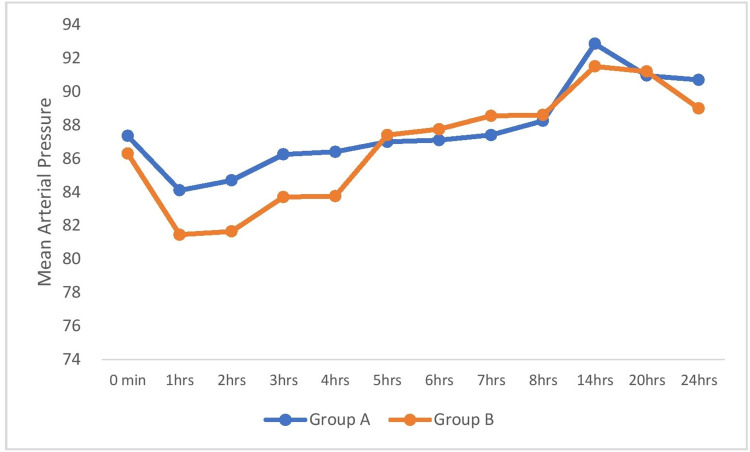
Comparison of mean arterial pressure (mmHg) among the groups.

Figure 3 shows the comparison of MAP among the groups across the time periods. There was statistically no significant difference in MAP between the groups at all the time periods.

**Figure 4:**
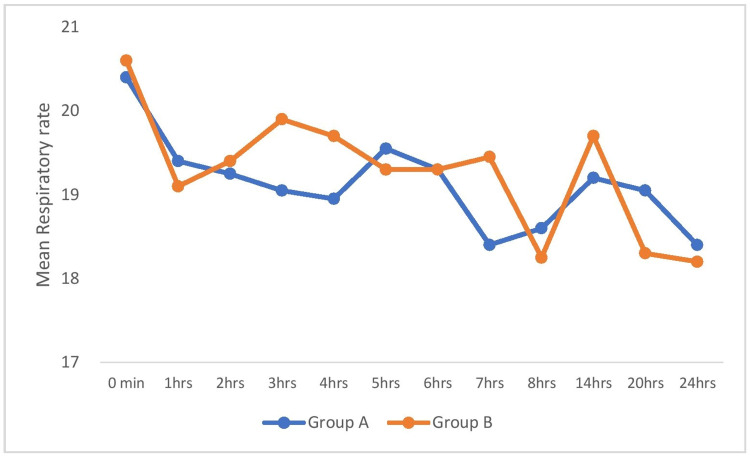
Comparison of respiratory rate (breaths per minute) among the groups.

Figure 4 shows the comparison of respiratory rate among the groups across the time periods. There was no significant difference in the respiratory rate between the groups at all the time periods.

Adverse effects like nausea, vomiting, dry mouth, allergic reaction, urinary retention, and respiratory depression were not found in either group.

## Discussion

Minimising pain after lumbar spine surgery is best achieved using a multimodal approach. Local anaesthetics, ranging from lidocaine and bupivacaine to the more recent ropivacaine, have been used as pre-emptive analgesics.

Local anaesthetic infiltration and instillation of drugs at the wound site is one of the simple and successful techniques for providing pain relief during the initial postoperative period after surgical procedures. There are numerous studies showing that infiltration of local anaesthetic bupivacaine into the surgical wound at the end of surgery decreases pain intensity, reduces the requirement of postoperative analgesics, and length of hospital stay. In the recent period, instillation of local anaesthetic solution intraperitoneally for postoperative analgesia has been extensively studied. It leads to early mobilisation, decreases the episodes of postoperative nausea and vomiting, and also reduces the use of parenteral opioids and non-steroidal anti-inflammatory drugs. In this study, we have compared the postoperative analgesic effect of infiltration versus that of instillation with bupivacaine for patients undergoing lumbar spine surgery under general anaesthesia.

In our study, the mean duration of analgesia was more in group A (instillation group) compared to group B (infiltration group) and the difference was statistically significant between both the groups. The findings in our study correlate to the study done by Bhattarai et al., who reported the duration of postoperative analgesia more in patients who received both intraperitoneal instillation and periportal infiltration undergoing laparoscopic cholecystectomy followed by patients in instillation group and shortest duration in infiltration group patients [14].

Another similar study, done by Prieto et al. compared the effectiveness of 7.5% ropivacaine instillation versus infiltration after radical mastectomy on 20 female patients [15]. They reported that infiltration or instillation of ropivacaine in the surgical wound preceding skin closure showed no statistically significant difference in postoperative pain (except for the requirement of rescue medication in the infiltrated group) showing the effectiveness of instillation compared to the infiltration of the drug at the surgical site, similar to our study.

In our study, the total amount of analgesia required was higher in the infiltration group compared to the instillation group and a statistically significant difference was noted between the groups. In concordance to our study, Jonnavithula [4] observed the role of wound instillation with bupivacaine (0.25%) through surgical drains for analgesic effects postoperatively in modified radical mastectomy and reported that cumulative rescue analgesic consumption and the number of demands for analgesia in the first 24 hrs, was significantly lower in the bupivacaine group compared with the saline group and control groups (P = 0.00).

Cherian and co-authors conducted a prospective randomized double-blind study to evaluate the effects of wound infiltration with bupivacaine after lumbar laminectomy and reported significant pain relief in the bupivacaine group compared to the placebo group [1]. In the postoperative period, the meantime before administration of the first dose of analgesic was more in the bupivacaine group compared to the placebo group.

Jonnavithula et al., in their study, evaluated the effects of wound instillation with bupivacaine through surgical drains in modified radical mastectomy and reported significantly less pain in the bupivacaine group compared to the control group [4]. The amount of rescue analgesia required was higher in the control group than in the bupivacaine group.

In our study, the mean NRS score was higher in the infiltration group compared to the instillation group till eight hours postoperatively and was comparable between the groups except in the thied hour and eighth hour. This could be due to the increased demands of rescue analgesia in group B patients owing to elevated NRS scores. After the 14th hour onwards, the mean NRS score was higher in group A compared to group B and statistically significant. This coincides with the first demand for rescue analgesia in group A patients. Hence, lower mean NRS score and prolonged duration of analgesia were observed in the instillation group compared to the infiltration group patients.

In a similar study, Jain et al. reported lower NRS score in laparoscopic cholecystectomy patients who received intraperitoneal instillation as well as trocar site infiltration compared to the patients who received trocar site infiltration only and the difference was statistically significant during the postoperative period between the groups (p<0.001) [16]. Another study done by Datta evaluated the role of intraperitoneal instillation of bupivacaine after laparoscopic cholecystectomy for post-operative pain management [17]. He reported that the mean NRS score of the control group at six and 12 hours postoperatively was found to be higher and statistically significant compared to the bupivacaine group. This showed the effectiveness of wound instillation for postoperative analgesia similar to our study. Gautam et al., in a prospective randomised clinical observational study in caesarean section, reported that 0.2% ropivacaine instillation with local site infiltration prolonged the duration of analgesia and low VAS score compared to the local site infiltration alone [18].

In our study, hemodynamic parameters were statistically non-significant between the groups. Prieto et al. compared the effectiveness of 7.5% ropivacaine instillation versus infiltration after radical mastectomy on 20 female patients divided into two groups [15]. They found no statistically significant difference in systolic and diastolic blood pressure between study groups. Similarly, Agrawal et al. evaluated postoperative pain relief with intra-peritoneal bupivacaine instillation in laparoscopic cholecystectomy and observed that hemodynamic parameters were statistically not significant between the groups [19].

Limitations

Pain perception is a subjective parameter and may vary from person to person. Pain on movement and during coughing was not tested. We did not study the difference of time to administer the rescue analgesia in the groups. Moreover, this study included a small patient population. Further studies can be done with a large sample size to clinically extrapolate the results.

## Conclusions

Pain management in the postoperative period has always been a major challenge for all surgeons and anesthesiologists, because of the expanding role of outpatient surgery and the need to facilitate hospital discharge earlier. In conclusion, instillation of local anaesthetic drugs in lumbar spine surgery provides effective analgesia in the postoperative period and thus its routine use is recommended. However, additional studies can be done with other agents. This technique of providing postoperative analgesia can be included in the armamentarium of multimodal analgesia.
